# Early Differences in Cognition Associated With Familial Longevity and ApoE Genotype Using Digital Technology

**DOI:** 10.1093/geroni/igaa057.2264

**Published:** 2020-12-16

**Authors:** Stacy Andersen, Mengtian Du, Nicole Roth, Mary Wojczynski, Bharat Thyagarajan, Thomas Perls, Stephanie Cosentino, Paola Sebastiani

**Affiliations:** 1 Boston University School of Medicine, Boston, Massachusetts, United States; 2 Boston University School of Public Health, Boston, Massachusetts, United States; 3 Washington University School of Medicine, St Louis, Missouri, United States; 4 University of Minnesota, Minneapolis, Minnesota, United States; 5 Columbia University Medical Center, New York, New York, United States; 6 Boston University, Boston, Massachusetts, United States

## Abstract

Merging digital technologies with neuropsychological testing allows for collection of novel metrics that may reveal early, subtle differences in cognitive functioning. We examined whether digital pen metrics from the Clock Drawing Test (CDT) differentiate healthy agers (i.e., individuals with familial longevity) from spouses and individuals by APOE genotype. We used generalized estimating equations adjusted for sociodemographics, familial longevity, and APOE genotype. Among 1974 participants with correct clocks (mean age 71±10 years), familial longevity was associated with better cognitive processing (i.e., shorter latencies/thinking time before cognitively demanding components) whereas the e4 allele was associated with smaller clock diameter and longer latencies. The e2 allele was negatively associated with total time and latencies. Therefore, digital metrics captured differences in cognitive processing among individuals with correct clocks and thus may be more sensitive than traditional scores. Additionally, familial longevity may confer cognitive advantages that are distinct from the risk/protection afforded by APOE genotype.

